# The association of low-level air pollution with birth weight in a register-based study: potential effects below WHO AQ guidelines

**DOI:** 10.1186/s12884-025-07219-6

**Published:** 2025-02-14

**Authors:** Isabell K. Rumrich, A. Korhonen, B. Forsberg, L. M. Frohn, C. Geels, J. Brandt, O. Hänninen

**Affiliations:** 1 Finnish Institute for Health and Welfare, Department of Public Health, Kuopio, Finland; 2 School of Pharmacy, University of Eastern Finland, Kuopio, Finland; 3 Department of Environmental and Biological Sciences, University of Eastern Finland, Kuopio, Finland; 4 Department of Public Health and Clinical Medicine, Umeå University, Umeå, Sweden; 5 Department of Environmental Science, Aarhus University, Roskilde, Denmark; 6 P.O. Box 95, Kuopio, FI-70701 Finland

**Keywords:** Air pollution, Birth weight, Low birth weight, Pregnancy, Prenatal, Register research

## Abstract

**Background:**

Air pollution exposure during pregnancy has been associated with adverse birth outcomes. Uncertainties remain about the effect at very low exposure levels. The aim of this study was to explore the association of maternal exposure to air pollutants during pregnancy at very low exposure levels with birth weight and estimate the health impact.

**Methods:**

The MATEX birth cohort (226,551 singleton births in 2012–2016) was linked with eight modelled air pollutants (PM_2.5_, PM_10_, PM_coarse_, NO_2_, NO_x_, CO, SO_2_, O_3_) at home address during pregnancy. Multiple regression was used to estimate the change in birth weight (in g) associated with individual-level mean exposure during pregnancy. We tested different adjustment models and conducted sensitivity analyses. We also estimated the potential number of low birth weight cases attributable to PM_2.5_ to quantify the public health issues at the prevailing low exposure levels.

**Results:**

PM_2.5_ was associated with the largest reduction of birth weight (-6.5 g per 1 µg/m^3^) followed by PM_crs_ (-4.9 g) and PM_10_ (-3.0 g). Among the gaseous pollutants the strongest reduction in birth weight was observed for NO_2_ (-0.8 g), followed by CO (-0.5 g), NO_x_ (-0.4 g) and SO_2_ (-0.2 g). On the contrary, O_3_ was associated with a modest increase in birth weight (+ 0.9 g). Effects on births weight were observed also below WHO guideline values. When accounting for the prevailing exposure levels in Finland, CO was associated with the biggest reduction in birth weight. The effect of PM_2.5_ exposure on birthweight corresponds to a loss of 30 g at mean exposure. Assuming a causal relationship, about 700 cases of low birth weight could be attributable to PM_2.5_ in Finland during the study period.

**Conclusions:**

No clear evidence on safe exposure level was found in this study. All pollutants were associated with reduced birthweight except ozone. Causality and confounding due to correlations warrant specific attention.

**Supplementary Information:**

The online version contains supplementary material available at 10.1186/s12884-025-07219-6.

## Background

Ambient air pollution is recognised as a risk for many adverse health outcomes, in particular premature mortality [1]. The effect of air pollution exposure during pregnancy on intrauterine growth has been studied for decades using weight-related outcomes at birth as endpoint [2]. Studies are characterised by great heterogeneity complicating assessment of causality, shape of the exposure-outcome relationship and identification of the most relevant pollutants [2].

Birth outcomes have been identified as an important indicator for prenatal development [3]. Prenatal development is also associated with later life health, i.e., the developmental origins of health and disease [4]. These associations are not fully understood and are largely omitted in environmental health assessments. The foetal and early postnatal life is characterised by the development of organ systems and tissues, which makes this period especially susceptible for exposures to chemical, physical and nutritional stress factors. Any effect of early exposures, potentially marked as adverse birth outcome, can persist and may lead to health consequences during adulthood [5–7]. Adverse effects in newborns after prenatal exposures, may occur even at doses clearly below no-effect levels in adults [8]. Birth weight is routinely measured at birth and is widely used as an indicator for sub-optimal prenatal development, e.g., defined as low birth weight (< 2,500 g) or small for gestational age. Even slight changes in birth weight, although they may not lead to low birth weight or small for gestational age, can be markers of disturbances during prenatal development. Thus, birth weight is an interesting endpoint to study the effects of prenatal exposure to ambient air pollution at levels as low as in Finland.

The understanding of the effects of air pollution on birth weight is limited. In a recent meta-analysis by Simoncic et al. [9], no statistically significant effects of NO_2_ were observed on birth weight. The studies included in the meta-analysis had mean NO_2_ exposure of between 20.5 and 37.9 µg/m^3^, below the WHO 24-hour mean guideline value, but still clearly higher than the concentrations prevalent in Finland. The evidence for an effect of particles on birth weight has been rated as inadequate for PM_2.5_ and PM_coarse_ and limited for PM_10_ in a recent meta-analysis [10]. The pooled estimates for exposure during the whole pregnancy were statistically significant with a reduction of 27.6 g (95% CI -48.5 g, -6.7 g; 15 studies) per 10 µg/m^3^ for PM_2.5_, a reduction of 8.7 g (95% CI -16.8 g,-0.48 g; 8 studies) per 10 µg/m^3^ for PM_10_ and a reduction of 8.8 g (95% CI -10.3 g,-7.3 g; 5 studies) per 10 µg/m^3^ for PM_coarse_ [10].

The current discussion on dose response shape and a potential threshold for effects is complicated by the multitude of pollutants (and their components) and health endpoints. Most epidemiological studies are conducted in areas with moderate to high air pollutant levels and very few studies are available with mean exposures below for example the WHO air quality guidelines [11]. Finland is a prime area to study air pollution health effects and contribute lacking data for the discussion of dose response at low exposure levels and a possible threshold due to the prevailing levels.

The general aim of this work was to establish the Finnish MATEX birth cohort air pollution data linkage for quantification of the effect of low-level air pollution on birth outcomes. The specific objectives were to (i) quantify the effect of air pollution during pregnancy on birth weight, and (ii) identify the most relevant air pollutants.

## Materials and methods

### Birth cohort

The MATEX birth cohort was established from the Finnish Medical Birth register and eligibility for this study was limited to birth between the years 2012 and 2016 for which additional area level socioeconomic covariates were available. The cohort has been described in detail elsewhere [12]. In short, the Finnish Medical birth register records information on all births with at least 22 gestational weeks or 500 g birth weight [13]. The information is collected from nurses and midwives on standardized forms during antenatal care visits. The register contains all basic information on the mother and her health, as well as the pregnancy, birth, and the child’s health up to seven days after births. Births with missing information on covariates, unclear sex and congenital anomalies, as well as multiple births, have been excluded (Supplemental Material Figure S1.). Geocoded residential address at birth were obtained from the Finnish Population Register.

### Air pollution and exposure assessment

Hourly air pollution concentrations at ground level were estimated for Northern Europe, including Scandinavia, Denmark, and Finland at 1 km x 1 km spatial resolution by Aarhus University. Average concentrations were calculated for 16 pollutants. Out of these 16 pollutants, seven plus PM_coarse_ (calculated as the difference between PM_10_ and PM_2.5_ concentrations) are covered here (PM_2.5_, PM_10_, PM_coarse_, NO_2_, NO_x_, CO, SO_2_, O_3_) for each pregnancy. Exposure to air pollutants was estimated at the home address during pregnancy by averaging hourly outputs of the air pollution model in the grid-cell where the home address was located. Specifically, the arithmetic mean was calculated by summing the hourly estimates and dividing them by the number of hourly observations for each pregnancy in the birth cohort.

The concentrations were modelled using the integrated multiscale air pollution model system, DEHM-UBM [14–16]. The intercontinental and regional background concentrations due to non-local natural and anthropogenic sources are calculated using a 3D chemistry transport model, the Danish Eulerian Hemispheric Model (DEHM), covering the Northern hemisphere and including complex atmospheric chemistry at a horizontal resolution down to 16.67 km x 16.67 km across the Nordic area [16]. These were then used as an input to the Urban Background Model (UBM) [14, 17], adding the local contribution from sources of primary anthropogenic pollutants up to 25 km from all grid points and calculating concentrations for the Nordic countries with a 1 km × 1 km resolution (using the ETRS89-LAEA grid definition).

### Endpoints and covariates

Birth weight was used as an endpoint as a continuous variable in grams.

Gestational age (in days or weeks) was included as a continuous variable in the regression model. Maternal age was included as a continuous variable in years and categorical variable. Sex of the child was defined as a binary variable (male, female). Season has been defined based on the birth month, winter defined as December to February, spring as March to May, summer as June to August, and autumn as September to November. The socioeconomic status of the mother was defined based on the Finnish national classification of occupations, categorized as upper white collar (upper-level employees with administrative, managerial, professional and related occupations), lower white collar (lower-level employees with administrative and clerical occupations), blue collar (manual workers) and others (farmers, self-employed, students, pensioners, no information). In addition, a category of “missing information” was added [18]. Parity was defined as nulli- or multiparous. Maternal pre-pregnancy BMI was calculated from the self-reported pre-pregnancy weight (kg) and height (m). Maternal smoking was based on self-reported smoking status during antenatal care visits, available as categories non-smoking, quitted smoking during the 1st trimester, and continued smoking after the 1st trimester. Fraction of postal code area occupants in the lowest national income quintile has been defined for the postal code area for the child’s address at birth. Income quintiles are defined based on the national annual distribution of that year. The fraction of the population that has at least secondary education (high school diploma) has been defined based on the highest academic degree held by each inhabitant in the postal code area [19].

### Statistical methods

We use a generalized linear model (R function glm) to predict the association of each pollutant (as continuous variable) with birth weight (in g, as continuous variable) in eight single-pollutant models. The associations were quantified for 1 µg/m^3^ increments as well as IQR increments. In addition to a crude, unadjusted model, we defined three adjustment models based on previously published literature and data availability (Table 1, Supplemental Material Table S1). The average exposure during the whole pregnancy was used as independent variable in the regression model.

**Table 1:** Adjustment models calculated for each of the eight pollutants (see Supplemental Material table S1)

Model	Covariates adjusted
Crude	None (univariate regression of birth weight by each pollutant)
Model 1	Gestational age (days), maternal age (years), sex, season (winter, spring, summer, autumn)
Model 2	Model 1 + maternal socioeconomic status, parity, maternal pre-pregnancy BMI, maternal smoking
Model 3	Model 2 + percentage of postal code occupants belonging to the lowest national income quintile, percentage of postal code area occupants having secondary education

### Sensitivity analyses

As sensitivity analyses, we explored potential changes due to exclusion of preterm births, modification by an exposure threshold, and extension of the cohort to include birth between 1991 and 2011 (beyond the area level SES variable data availability). In the first sensitivity analysis we excluded all newborns born before 37 weeks of gestation assuming that the gestational age, especially in preterm birth, is the main determinant of (low) birth weight and thus potentially masking other, weaker determinants, such as air pollution exposure. Sensitivity to threshold was tested by excluding the lowest proportion of exposure (mean of 5th percentile minus minimum) in the regression model, assuming that the exposure misclassification is highest in the tails. We also considered an additional adjustment model including a national region indicator, which was defined using the first digit of the postal code at residential address. We also tested for sensitivity to alternative definition of the maternal age variable (categorical instead of continuous, Supplemental Material, Table S5) and gestational age (weeks instead of days). We also stratified the analyses by sex to explore differences in the sensitivity of the sexes to exposure to air pollution. We tested for effects of air pollution below the WHO AQ guideline values [20] by restricting the study population to pregnancies exposed only to exposure lower than the guidelines (Supplemental Material, Sect. 7).

### Relative risks for low birth weight and impact assessment

To quantify the hypothetical public health impact of air pollution at prevailing low exposure levels, we calculated hypothetical no-exposure birth weights. We subtracted the single-pollutant Model 3 (see Table 1) impact for each cohort member to represent the counterfactual birth weight on individual level (Eq. 1):

1 $$\eqalign{ B{W_{est}} & = B{W_{obs}} + \Delta BW \cr & \quad = B{W_{obs}} + {\beta _{model\>3}} \times C \cr}$$

where *BW*_*est*_ is the hypothetical counterfactual birthweight, *ΔBW* is the estimated change in birthweight *β* is the slope coefficient for air pollutants in Model 3 (Table 2; Supplemental Material, Table S4) and *C* the exposure of the mother during the pregnancy. The newborns that were lifted above the low birth weight threshold (2500 g) were considered as attributable cases. Using the estimated attributable cases in Model 3 we calculated the risk ratio (RR) for low birth weight at the observed mean PM_2.5_ exposure level (4.7 µg/m³) and scaled it further per 10 µg/m³ for wider comparability between studies and endpoints.

**Table 2:** Change in birth weight (g) per IQR in air pollution (pregnancy average) with all p values < 0.001

	IQR	Crude model		Model 1^a^		Model 2^b^		Model 3^c^	
	µg/m^3^	beta	±se	beta	±se	beta	±se	beta	±se
**PM**_**2.5**_	2.07	-18.9	1.6	-41.5	1.4	-16.9	1.3	-21.1	1.4
**PM**_**10**_	4.28	-22.3	1.5	-41.5	1.2	-17.1	1.2	-20.5	1.3
**PM**_**crs**_	2.44	-23.5	1.4	-39.6	1.2	-16.5	1.2	-19.0	1.2
**NO**_**2**_	13.87	-22.3	1.3	-39.4	0.9	-16.9	0.9	-19.0	1.1
**NO**_**x**_	21.54	-17.2	1.1	-30.8	0.9	-13.1	0.9	-14.9	0.9
**CO**	30.00	-22.8	1.5	-43.8	1.2	-21.0	1.2	-24.6	1.2
**SO**_**2**_	21.47	-16.3	1.1	-26.4	0.9	-9.5	0.9	-10.1	0.9
**O**_**3**_	10.93	26.7	1.4	41.3	1.2	19.5	1.2	21.0	1.2

se: standard error

^a^ adjusted for gestational age (days), maternal age (years), sex, season

^b^ adjusted for Model 1 and maternal socioeconomic status, parity, maternal pre-pregnancy BMI, maternal smoking

^c^ adjusted for Model 2 and lowest Income quintile (postal code level), fraction of secondary education (postal code level)

### Ethics approval and register data permit

In accordance with the Finnish Medical Research Act (1999/488) the MATEX study including the birth cohort identified from the Medical Birth Register has been evaluated and approved by the official ethics committee of the Northern Ostrobothnia Hospital District (EETTMK 44/2016; issued 18th of April, 2016). The right to use register data held by the Finnish Institute for Health and Welfare was granted under document number THL/984/6.02.00/2020 (issued 17th of March, 2020). Due to the full register-based design of the study, no informed consent was required from the study participants according to the Finnish Personal Data Act 1050/2018.

## Results

A total of 226,551 mother-child pairs were included in the main analyses (Supplemental Material, Figure S1). The average maternal age was 29.9 (SD 5.3, min 14 years, max 55 years) years and most mothers were nulliparous and non-smoking (Table 3). The cohort consists of slightly more boys (50.7%), the average birth weight was 3537 g and the average gestational age was 39 weeks + 6 days.

**Table 3:** Descriptives of the MATEX cohort (2012–2016, singletons)

Variable	Count (%)	Mean (SD)
Total births in the current cohort	226,551 (100%)	
**Mother**		
Age (years)		29.9 (5.3)
Pre-pregnancy BMI		
Underweight	8207 (4%)	
Normal weight	139,451 (62%)	
Overweight	49,955 (22%)	
Obese	28,938 (13%)	
Multiparous	91,048 (40%)	
Socioeconomic status		
Upper white collar	24,712 (11%)	
Lower white collar	48,883 (22%)	
Blue collar	18,769 (8%)	
Other	26,226 (12%)	
Missing	107,961 (48%)	
Smoking during pregnancy		
Quit during 1st trimester	15,879 (7%)	
Continued after 1st trimester	19,124 (8%)	
**Child**		
Season of birth		
Spring (March – May)	53,375 (24%)	
Summer (June – August)	57,430 (25%)	
Autumn (September – November)	59,860 (26%)	
Winter (December – February)	55,886 (25%)	
Sex (male)	114,051 (50%)	
Birth weight (g)		3507.9 (482.1)
Gestational age (days)		278.8 (10.9)
Preterm birth (< 37 weeks)	8933 (4%)	
Low birth weight (< 2500 g)	6002 (3%)	
**Postal Code area level confounder**		
In lowest income quintile	55,764 (24%)	
At least secondary education	164,969 (71%)	
**National region**		
0	66,064 (29%)	
1	14,168 (6%)	
2	27,016 (12%)	
3	26,449 (12%)	
4	17,511 (8%)	
5	8310 (4%)	
6	20,575 (9%)	
7	9367 (4%)	
8	12,895 (6%)	
9	24,916 (10%)	

**Fig. 1:**
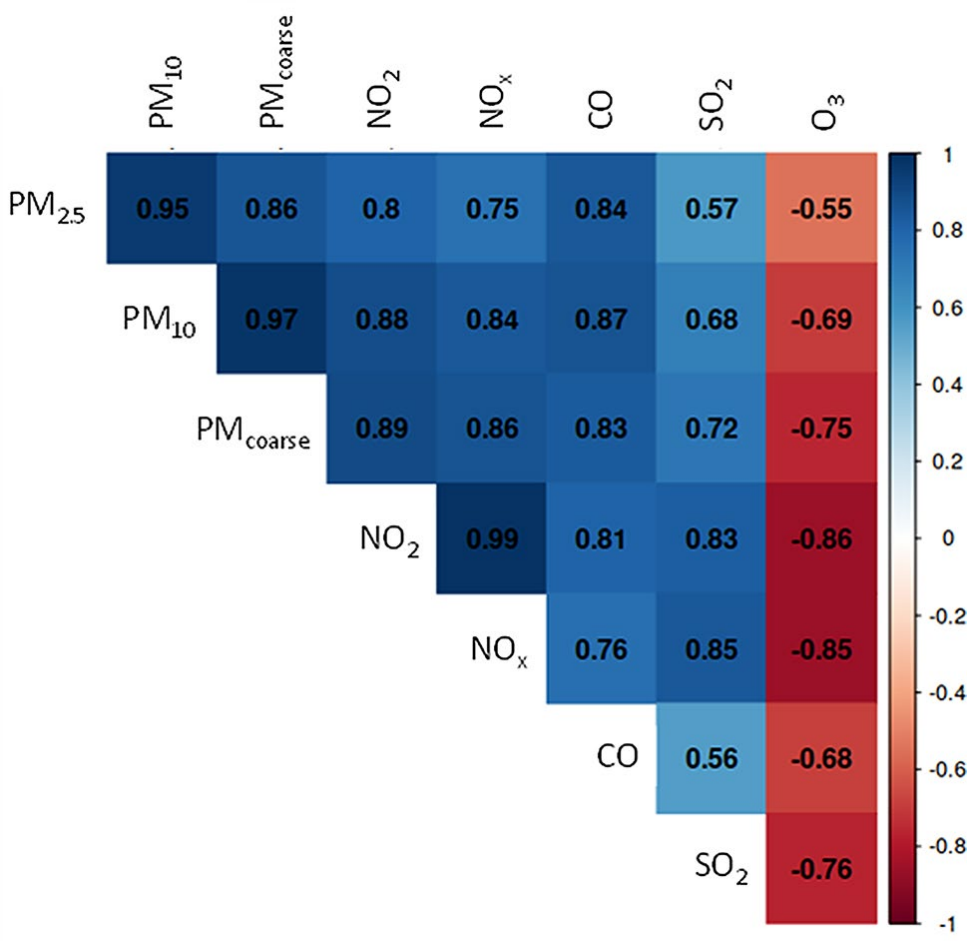
Pearson correlation matrix for the average air pollution exposures during pregnancy at residential address

All pollutants, estimated as outdoor concentrations at the residential address at birth, were highly correlated (Pearson correlation coefficient > 0.5). For O_3_ the correlation coefficients were of similar absolute magnitude, but negative (Fig. 1). In comparison with all the other covariates, the pollutant correlations were clearly the highest. Especially high correlations were among the particulate matter fractions PM_2.5_, PM_10_ and PM coarse, and between NO_2_ and NO_x_, both groups representing overlapping components and emission sources.

**Fig. 2:**
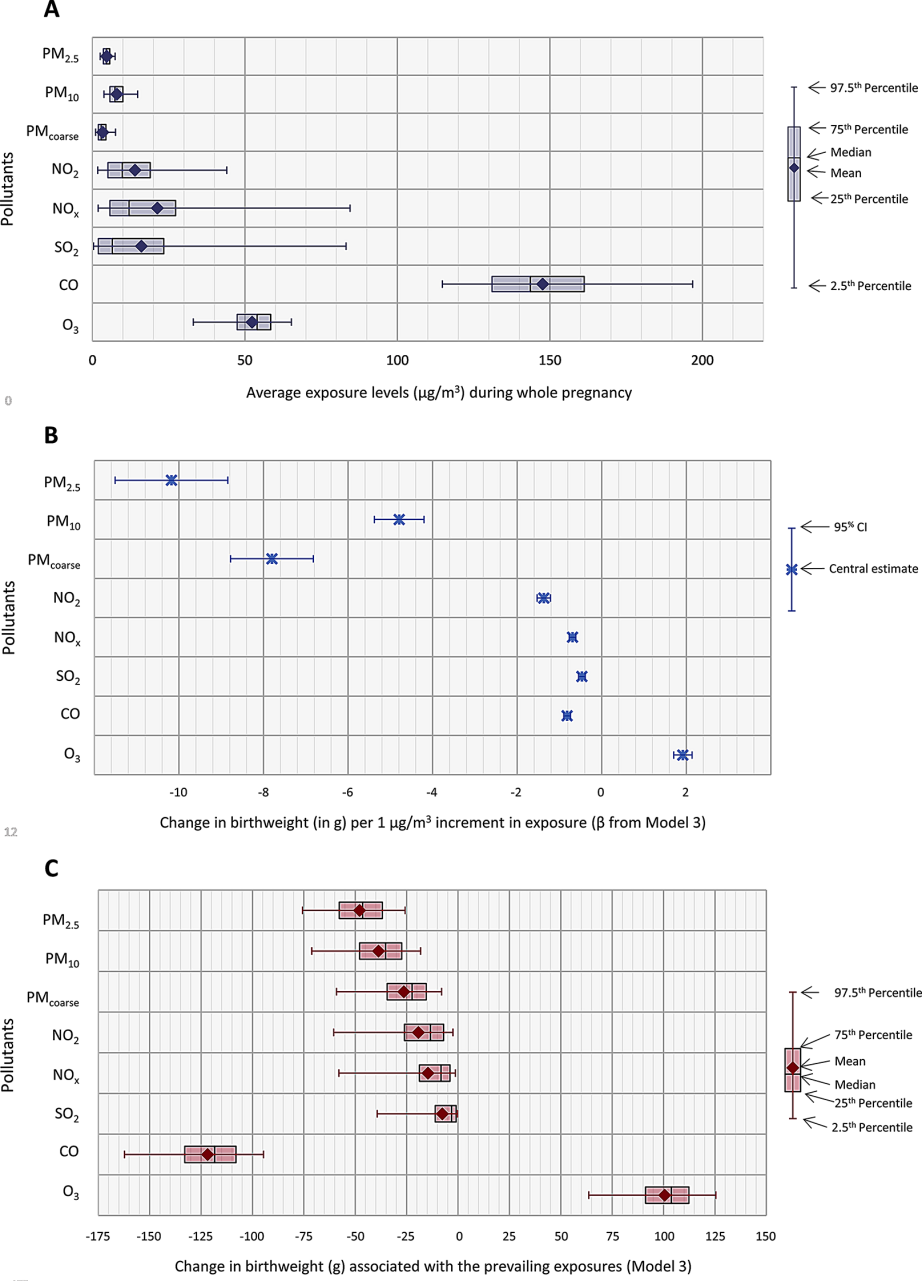
(**A**) Distribution of air pollutant exposures among Finnish pregnant women. (**B**) Mean change in birth weight and 95% CI (in g per 1 µg/m^3^ increment in exposure) from Model 3; (**C**) Impact assessment of changes in birth weight (in g) in Finnish newborns between 2012 and 2016

All exposure levels in Finland were relatively low in comparison with similar studies in other areas (Fig. 2A; Supplemental Material, Table S2). On average, exposure was higher in Southern part of the country and in urban areas, only O_3_ being higher in rural areas. A more detailed spatial analysis has been presented elsewhere [21].

### Changes in birth weight

Exposure levels to all studied air pollutants were associated with the birth weight among this Finnish cohort with high statistical significance (p-value < 0.001). The observed associations of the eight pollutants with birth weight were consistent over the four models calculated. Largest differences between the coefficients were observed for Model 1 (M1), which suggested larger effect estimates for all pollutants. Models 2 and 3 yielded similar air pollutant associations concerning both direction and magnitude of the coefficients (Table 2).

In general, particles were associated with lower birth weight than gaseous pollutants (Fig. 2B; Table 2). Among the studied particle fractions, PM_2.5_ was associated with the strongest reduction in birth weight (-21.1 g per IQR (2.07 µg/m^3^)) followed by PM_10_ (-20.5 g per IQR (4.28 µg/m^3^)) and PM_coarse_ (-19.0 g per IQR (2.44 µg/m^3^)). Among the gaseous pollutants the strongest reduction in birth weight was observed for CO (-24.6 g per IQR (30.0 µg/m^3^)), followed by NO_2_ (-19.0 g per IQR ((13.87 µg/m^3^)), NO_x_ (-14.9 g per IQR (21.54 µg/m^3^)) and SO_2_ (-10.1 g per IQR (21.47 µg/m^3^)). On, the contrary, O_3_ was associated with a modest increase in birth weight (21.0 g per IQR (10.93 µg/m^3^)).

When stratifying the analyses by sex, a stronger reduction in birth weight associated with exposure to air pollution, was observed for girls compared to boys (Table 4).

**Table 4:** Main analyses (adjustment model 3), stratified by sex. Changes in birth weight (in g) per IQR increment in exposure

	IQR	Whole cohort^a^		Boys only^b^		Girls only^b^	
	µg/m³	beta	se	beta	se	beta	se
PM_2.5_	2.07	-21.1	1.41	-18.5	1.88	-25.4	1.90
PM_10_	4.28	-20.5	1.28	-17.6	1.71	-23.5	0.17
PM_crs_	2.44	-19.0	1.22	-16.1	1.61	-21.0	1.63
NO_2_	13.9	-19.0	1.11	-16.2	1.39	-21.4	1.39
NO_x_	21.5	-14.9	0.86	-12.7	1.08	-16.4	1.08
CO	30.0	-24.6	1.20	-21.3	1.80	-27.6	1.80
SO_2_	21.5	-10.1	0.86	-7.9	1.07	-11.8	1.29
O_3_	10.9	22.1	1.20	17.2	1.53	22.7	1.64

^a^ adjusted for gestational age (days), maternal age (years), sex, season, maternal socioeconomic status, maternal pre-pregnancy BMI, maternal smoking, lowest income quintile (postal code level), fraction of secondary education (postal code level)

^b^ adjusted as model 3 (see ^a^) minus sex

We did not observe indication for a non-linear relationship between the pollutants and birth weight in categorical quartile analyses (Supplemental Material, Figure S2, Table S3). The only exception was SO_2_ with non-linearity in the categorical analyses of quartiles 2 and 3 in reference to quartile 1.

In the sensitivity analyses excluding preterm births, the risk estimates for term births are slightly smaller than those for the total cohort. However, the differences are so small, that they can be considered practically similar. The risk estimates for only preterm birth are not shown as limited statistical power led to unreliable results. Exclusion of the lowest exposure percentiles did not result in a change of the observed regression coefficients (Supplemental Material, Table S4). Inclusion of an indicator variable for region resulted in lower risk estimates (Supplemental Material Table S4), possibly due to sparse data bias or non-positivity bias.

In addition, we tested for sensitivity of the adjustment models to variable definition. Inclusion of maternal age as a categorical variable (Supplemental Material Table S5) and gestational age in weeks instead of days, did result in similar risk estimates (Supplement Material S6).

Overall, consistent results were observed at internationally regarded very low exposure levels. Detailed analyses presented in the supplemental material did provide support for adverse effects below the current WHO AQ guideline values. The risk estimates differ from those of the total population: While the effect seems smaller for PM_2.5_, it is increased for NO_2_ and PM_10_ (Supplemental Material, Table S7). Applying categorical quartile analyses using the lowest quartile as a reference also demonstrated changes in birth weight at very low exposure levels (Supplemental Material Figure S1, Table S3). Exclusion of the lowest 2.5% of the exposure range did not affect the regression coefficients (Supplemental Material, Table S4). Exclusion of up to the 10th percentile resulted in no change in the estimate (data not shown).

### Impact assessment

To be able to compare the importance of observed effect of the different pollutants we scaled them to the average exposure during pregnancy in the study population. At the population level, CO is on associated with the highest reduction in birth weight with − 121.0 g (Fig. 2C). For exposure to PM_2.5_ the second highest reduction was observed with − 47.8 g at 4.7 µg/m^3^ average exposure. Ozone was associated with an increase of birth weight of about 100.4 g at the mean exposure level of 52.3 µg/m^3^ (Supplemental Material Table S8).

The observed risk of low birth weight (< 2,500 g) was 2.6%. Based on the M3 regression (Tables 1 and 2) estimates for the pollutants, 714 cases of low birth weight were associated with PM_2.5_, yielding a relative risk at the prevailing exposure distribution of 1.14, or RR = 1.29 per 10 µg/m^3^. The relative risk estimates are rather highly dependent on the choice of the model (Table 5).

**Table 5:** Observed incidence of low birth weight (birth weight < 2,500 g), estimated attributable cases for PM_2.5_ at the prevailing exposure distribution, and corresponding relative risks at prevailing exposure (4.7 µg/m^3^) and per 10 µg/m^3^

	Cases	PM_2.5_ attributable cases	RR at average PM_2.5_ exposure level (4.7 µg/m^3^)	RR at 10 µg/m^3^
Observed	6,002	n/a		
Model 1 *		1334	1.29	1.61
Model 2 *		571	1.11	1.22
Model 3 *		714	1.14	1.29

* adjustment model from the regression analysis (see Table 1)

## Discussion

In this study we used a register-based approach to estimate the association of modelled air pollution exposure during pregnancy with birth weight, with potentially differential effects in male and female infants. The air pollution levels for the MATEX birth cohort were well below EU limit values and even largely lower than the health-based guidelines. Nevertheless, we observed an association with a reduction of birth weight up to 6.5 g per 1 µg/m^3^ (corresponding to 21.1 g reduction per IQR) increase in PM_2.5_. SO_2_ was associated with the smallest reduction in birth weight (3.7 g per IQR (21.5 µg/m^3^) and 0.5 g per 1 µg/m^3^ increment).

The high correlation of air pollutants (Fig. 1) complicates the identification of the causative exposure(s). The observed associations may be influenced by one specific pollutant or a mixture of several pollutants. Thus, it is not possible to sum the reduction in birth weight across the pollutants, but rather interpret each observed effect with the pollutant as indicator. However, as the numerical range of exposures differ substantially between the pollutants, PM_2.5_ having the lowest range with mean at 4.7 µg/m^3^, to consider the impact of each pollutant to the outcome the ranges and the slopes must be considered. Even when taking average exposure into account, PM_2.5_ seems to be the most relevant particulate matter size fraction (Fig. 2C), but it is superseded by more than a factor of two by the impact of CO. This finding is highly unexpected, even though the known carboxyhaemoglobin binding mechanistically would be consistent with reduced metabolism and growth. The impact of CO on birth weight is very high. There is a considerable contribution of CO from all combustion sources suggesting that it is an excellent indicator of the overall effects of combustion related air pollution or that there are some unexpected confounding issues to be solved. CO originates from combustion processes with catalysts removing only a fraction. Shipping, other mobile sources, and power plants contribute greatly. In comparison to PM_2.5_ and NO_2_, which are also emitted by combustion processes, it has a rather long half time of about a month. Thus, CO concentrations are greatly influenced by long range transport with peaks around direct combustion sources. In Finland, residential wood combustion is an important source of combustion emissions [22]. It will be important to consider whether the high impact of CO could indicate harmful effects of residential wood combustion, mediated by CO directly, or by other components that CO would be an indicator of.

There are contributions to the particle concentration from local emission sources like traffic and residential wood combustions, but also a significant contribution from similar sources further away, through long-range transport [16, 23]. The composition of particle varies across season and region. During colder weather, small scale wood combustion is increased for additional heating. Sea salt and sulphate from shipping emissions content in particles is higher in coastal regions compared to inland. In addition, traffic emissions are higher in high-traffic areas, i.e., dense urban areas, compared to rural regions. Secondary aerosols are highly variable and are formed from anthropogenic and biogenic gases emitted from sources ranging from local to remote sources [24]. Similarly, nitrogen oxides are emitted from traffic, however power plants, industry and mobile sources and ships greatly contribute, potentially also via long-range transport. Sulphur dioxide is produced by power plants, regional heating and industries, all point sources by nature. Up until 2015, where international regulations of sulphur content in marine fuels were introduced, shipping was a major source of sulphur dioxide in the atmosphere.

Ozone was associated with inversed effects, on average an increase of birth weight by about 100 g. As ozone had negative correlation with all the other pollutants, our interpretation is that this finding is an artefact, and that ozone is not actually a protective exposure against low birth weight. Interestingly the negative impact of CO is of same magnitude as the impact of ozone. These surprising findings have to be elaborated in the follow-up, when applying multipollutant models. Ozone is generated by photochemical reactions in the atmosphere and has highest concentrations in rural background areas while being consumed by air pollution chemistry in urban and other polluted areas, leading to the observed and expected negative correlation.

For particles, their size is an important determinant of dispersion, infiltration, respiratory tract uptake and thus dose. Bigger particles tend to be removed by deposition, infiltration and captured in the extrathoragic part of the respiratory tract, reducing penetration deeper into the lung. Particles are likely to be the most heterogeneous pollutant in terms of sources. In Finland, roughly half of the PM_2.5_ is expected to originate from long-range transport. National emissions are rather equally divided between residential wood combustion and traffic emissions, which further originate partly from the tailpipe emissions, partly from the tyre-road surface interface and brakes.

Besides carbon monoxide, SO_2_ is also not as well studied. The observed association with birth weight was modest with a reduction of 3.7 g per IQR (21.5 µg/m^3^) or 0.2 g per 1 µg/m^3^. The SO_2_ exposures indicated a very skewed distribution with relatively low median levels and substantially higher outliers, presumably located close to these industrial sources.

In this study we interpreted the PM_2.5_ exposure as the key exposure in the impact assessment, however based on the data shown in Fig. 2C, alternative interpretations would be that CO would be the key pollutant, or ozone would have a protective effect. The other observed associations are due to the correlations of the pollutants. The effects may even be due to the pollutant mixture.

### Previous studies

The association between prenatal ambient air pollution exposure and birth weight has been investigated in numerous studies with the evidence being rated from insufficient to limited, depending on the pollutant [10]. Overall, there are substantial methodological differences between published studies, such as exposure estimates (modelled vs. monitoring data), adjustment factors included in the regression, assumption about exposure-response shape (linear vs. non-linear) and study population (e.g., exclusion of preterm births; register-based design vs. questionnaire data). In addition, the study sizes vary greatly from e.g. about a thousand births [25] to several million births [26]. The published studies cover a wide range of air pollution levels, e.g. studies focused on NO_2_ range in mean exposure from 13.6 µg/m^3^ [27] to 52.7 µg/m^3^ [28], studies focused on PM_10_ from 10.7 µg/m^3^ [29] to 50.76 µg/m^3^ [27]; studies focused on PM_2.5_ from a median of 7.45 µg/m^3^ [25] to a mean of 27.49 µg/m^3^ [27].

Residential outdoor exposure to air pollution during pregnancy was consistently associated with a reduction in birth weight, except of exposure to ozone, which was associated with an increase in birth weight. Per 1 µg/m^3^ increment in exposure, PM_2.5_ was the most relevant pollutant, followed by PM_coarse_. From the gaseous pollutants NO_2_ was associated with the biggest reduction in birth weight per 1 µg/m^3^ increment in exposure. However, when considering the prevailing average exposure levels in Finland, CO was associated with the biggest reduction in birth weight. The large negative effect of CO requires more detailed evaluation in the follow-up. For the classical pollutants (particles and nitrogen-oxides) the observed slopes were higher here than in the studies reported in the literature. This study showed, that modelled low-level air pollution is associated with reduction in birth weight, strengthening the evidence that there is no safe level or threshold for exposure to air pollution in pregnant women. The observed changes in risk estimates between the total birth-cohort and the pregnancies restricted to below the WHO guideline values (Supplemental Material, Table S7) may be due to selection bias of pregnancies from generally cleaner environments, as well as uncertainties in the dose-response from reduced study power. There is strong evidence for the other studied pollutants for clear adverse effects of exposure below the WHO guidelines, based on the subpopulations and quartile analyses (Supplemental Material, Table S3). The evidence for no threshold for PM2.5 effects is more limited based on the decrease in risk estimate in the subpopulation exposed to below 5 µg/m3 and the absence of statistically significant effect when comparing the 2nd exposure quartile with the 1st quartile (Supplemental Material, Figure S2, Table S3).

The estimates of reduction in birth weight after prenatal exposure to ambient air in our current study are higher than those previously published. A previously published meta-analysis reports a reduction in birth weight by -23 g (95% CI -1.4, -45.5 g) for each 10 µg/m^3^ increment in PM_2.5_, a reduction of -17 g (95% CI -13.3, 20.2 g) for each 20 µg/m^3^ increment in PM_10_, and − 28 g (95% CI -11.5, 44.8) reduction for each 38.25 µg/m^3^ increment in NO_2_ [2]. No statistically significant association with CO, SO_2_ or O_3_ was found in the meta-analysis of 4, 3 and 7 studies, respectively [2]. In some studies, NO_2_ was not statistically significantly associated with changes in birth weight [27, 28]. For PM_10_ a differential association in boys and girls was reported. While no statistically significant association was observed in girls, each 10 µg/m^3^ increment in PM_10_ exposure was associated with a loss of 15.4 g in birth weight in boys [28]. In a Swedish study exhaust PM was associated with a strong decrease in birth weight of 7.5 g (95% CI -12.0 g, -2.9 g) per 209 ng/m^3^ increment, while no statistically significant association between birth weight and ozone was observed in a linear model [30]. A recent study of almost 500,000 births reports stronger decrease in birth weight for preterm births with about − 37 g (95% CI -61 g, -14 g) per 5 µg/m^3^ increase in PM_2.5_ and no association with NO_2_. Exposure to PM_2.5_ (per 5 µg/m^3^) or NO_2_ (per 10 ppb) was associated with 8 g (95% CI -16 g, -1 g) and 11 g (95% CI -15 g, -9 g), respectively, reduction in birth weight in term births in a linear fashion [31]. In contrast, Fu and coworkers utilizing the UK Biobank database report indication for a non-linear association between air pollutants and birth weight. They observed a strong decrease in birth weight up to a certain exposure level, i.e., 10.74 µg/m^3^ PM_2.5_, 16.06 µg/m^3^ PM_10_, 25.58 µg/m^3^ NO_2_ and 39.88 µg/m^3^ NO_x_ [32]. These turning points of the associations are well above the 75th percentile of exposure levels observed in our study. Thus, our observed effect estimates may be substantially higher than in other reported studies due to the shape of the exposure response curve and the stronger effect at lower concentrations.

Previous studies indicated differential sensitivity to prenatal exposure to air pollution by the offsprings sex, with male offspring suggested to be more vulnerable [28, 33, 34]. This contrasts with our results showing higher risk estimates in female offsprings. One Japanese study reported decreased sex ratio during higher pollution seasons, indicating a loss of male pregnancies during early pregnancy [35]. It can be hypothesized that the observed higher effect in girls could be partly attributable to depletion of susceptibles in the male offspring population.

Differences in the observed associations between air pollutants and birth weight may be due to several reasons, which are not mutually exclusive. For example, Sellier and colleagues demonstrated that the strength and magnitude of association with birth weight is sensitive to the choice of exposure model [36]. Another possibility is that the exposure-response curve across the whole exposure range investigated in the past, is not linear and at the lower end of the exposure range the effect is larger per unit increment that at higher exposure levels. For example Vodonos et al., [37] showed that in a meta-regression analyses at lower exposure range the percent change in deaths per 1 µg/m^3^ slopes up. While the endpoint was different, it remains interesting to consider effects of air pollution at low levels in more detail when more studies like this become available. Furthermore, the sources and composition of particles differ across time and space, which can lead to differences in toxicity and thus observed effects [38]. It has been discussed that birth weight is multi-factorial and that the effect of air pollution may not be evenly distributed across the population but varying with co-exposures and other factors [39]. This indicates that it cannot be expected that same effects are observed across different populations.

### Exposure misclassification

Previous research showed that the exposure assessment methods had significant impact on the estimates of health effects [36]. Independent of the endpoint studied, there is an underlying discussion about the shape of the exposure response relationship and a potential threshold when studying the health effects of ambient air pollution (e.g [40]). The impact of exposure estimation error on the assumed exposure-response relationship has been discussed by Cox [41]. They conclude that the error in estimated PM_2.5_ concentration in comparison to observed concentration was sufficient to smooth the exposure-response relationship to approximately linear. and we assume no threshold without threshold. We conducted a sensitivity analysis assuming a threshold at 2.5th percentile of the exposure, which did not lead to a change in the estimate for beta for any of the air pollutants (Supplemental Material, Table S4), as well as quartile analyses (Supplemental Material, Tables S3, Figure S2) and sensitivity analyses in the subpopulation exposed to air pollution levels below the WHO AQ guidelines (Supplemental Material, Table S7).

In this study, we were not able to take residential mobility into account, but we assumed the address at birth as address throughout pregnancy. This can lead to some exposure misclassification. Previous studies found little impact of taking address changes during pregnancy into account [42, 43]. However, these studies utilised monitored air pollution data with bigger spatial aggregation than in our current study. In our follow-up work we showed that the impact of residential mobility on exposures was only minor [44]. However, we also observed rather substantial risk of bias towards zero due to exposure misclassification during pregnancy [44].

### Strength and limitations

The full register-based design of this study comes with advantages and shortcomings. An advantage is that virtually the whole population is covered, leading to practically complete population representativeness. In addition, all information is routinely collected by nurses and midwives during antenatal care visits, thus minimising the risk for recollection bias and providing standardized data collection methods. However, personal information of the mother, such as smoking habits and occupation, which has been used to defined socioeconomic status in this current study, are self-reported. It cannot be excluded that the mother was not honest in her response. Furthermore, the full register-design limited the availability of confounder variables, potentially allowing residual confounding. The Finnish gradient of population density and urbanity from south to north comes with differences in lifestyle and exposures. We were able to adjust our estimates for maternal smoking and socioeconomic status of the mother. These are highly correlated with general lifestyle in the Finnish population. They have been earlier shown to be reliable makers for unaccounted lifestyle factors [39]. Paternal smoking and other second-hand smoke exposure during pregnancy has been shown to be associated with low birth weight, thus being an important adjustment factor. However, this information was missing in our study, possibly confounding our results [45].

Air pollution exposure at the home address was modelled on a 1 km x 1 km grid. The DEHM-UBM air quality model has been shown to be as reliable as air quality models generally are [16]. Nevertheless, there is a risk for exposure misclassification, which can bias the estimates in both directions, potentially leading to over- or underestimation of the effect [46]. Further, the residential outdoor exposure estimate does not account for mobility of the subjects between their homes, work- or study locations and other places being part of their daily activities. Most importantly, the outdoor levels do not account for infiltration of the ambient pollution, taking place when the air passes through the building envelope, structures, and ventilation systems. Indoor exposure studies have well demonstrated that especially particulate matter is filtered, but similar effects have been observed for nitrogen oxides and ozone, too.

### Robustness of results and generalizability

In our study we had to exclude about 9% of singletons births without birth anomalies in the dataset due to missing co-variates or exposure (Supplemental Material, Figure S1). While this raises the risk for selection bias, we did not observe big differences in covariate distributions between the analyzed cohort and the total cohort (data not shown).

The results were consistent across different adjustment models and sensitivity analyses. Testing for the sensitivity for the exclusion of preterm birth, as gestational age is the strongest predictor for birth weight, did not significantly change our results. Similarly, excluding the fraction of population with the highest exposure misclassification, e.g., the lowest 2.5th percentile, did not change the estimates, indicating the lack of a threshold.

## Conclusions

In this study, we quantified the associations of low-level air pollution with birth weight in a Finnish register-based birth cohort. Residential outdoor exposure to air pollution during pregnancy was consistently associated with lower birth weight, except of exposure to ozone, which was associated with an increase in birth weight. Per 1 µg/m^3^ increment in exposure, PM_2.5_ was the most relevant pollutant, followed by PM_coarse_. From the gaseous pollutants NO_2_ was associated with the biggest reduction in birth weight per 1 µg/m^3^ increment in exposure. However, when considering the prevailing average exposure levels in Finland, CO was associated with the biggest reduction in birth weight. The large negative effect of CO requires more detailed evaluation in the follow-up. For the classical pollutants (particles and nitrogen-oxides) the observed slopes were higher here than in the studies reported in the literature. This study showed that modelled low-level air pollution is associated with changes in birth weight even below current WHO AQ guideline values.

## Electronic supplementary material

Below is the link to the electronic supplementary material.

Supplementary Material 1

## Data Availability

Data may be obtained from a third party and are not publicly available. The Finnish Institute of Health and Welfare is controller of the Medical Birth Register. Data may be obtained from the register controller.
