# Supplementary material for: The association of low-level air pollution with birth weight in a register-based study: potential effects below WHO AQ guidelines
Source: BMC Pregnancy Childbirth. 2025 Feb 14;25:162. doi: 10.1186/s12884-025-07219-6 (PMC11829368; doi:10.1186/s12884-025-07219-6)
Supplement: Supplementary file 1 — Supplementary Material 1 [file 12884_2025_7219_MOESM1_ESM.docx]

**Supplemental Material**

**The association of low-level air pollution with birth weight in a register-based study:**

**Potential effects below WHO AQ Guidelines**

Rumrich IK*^1,2^, Korhonen A^1,3^, Forsberg B^4^, Frohn LM^5^, Geels C^5^, Brandt J^5^, Hänninen O^1^

^1^ Finnish Institute for Health and Welfare, Department of Public Health, Kuopio, Finland

^2^ University of Eastern Finland, School of Pharmacy, Kuopio, Finland

^3^ University of Eastern Finland, Department of Environmental and Biological Sciences, Kuopio, Finland

^4^ Umeå University, Department of Public Health and Clinical Medicine, Umeå, Sweden

^5^ Aarhus University, Department of Environmental Science, Roskilde, Denmark

Corresponding author: Isabell Rumrich ([isabell.rumrich@thl.fi](mailto:isabell.rumrich@thl.fi)), P.O. Box 95, FI-70701 Kuopio, Finland

Abstract

**Background:** Air pollution exposure during pregnancy has been associated with adverse birth outcomes. Uncertainties remain about the effect at very low exposure levels. The aim of this study was to explore the association of maternal exposure to air pollutants during pregnancy at very low exposure levels with birth weight and estimate the health impact.

**Methods:** The MATEX birth cohort (226,551 singleton births in 2012-2016) was linked with eight modelled air pollutants (PM_2.5_, PM_10_, PM_coarse_, NO_2_, NO_x_, CO, SO_2_, O_3_) at home address during pregnancy. Multiple regression was used to estimate the change in birth weight (in g) associated with individual-level mean exposure during pregnancy. We tested different adjustment models and conducted sensitivity analyses. We also estimated the potential number of low birth weight cases attributable to PM_2.5_ to quantify the public health issues at the prevailing low exposure levels.

**Results:** PM_2.5_ was associated with the largest reduction of birth weight (-6.5 g per 1 µg/m^3^) followed by PM_crs_ (-4.9 g) and PM_10_ (-3.0 g). Among the gaseous pollutants the strongest reduction in birth weight was observed for NO_2_ (-0.8 g), followed by CO (-0.5 g), NO_x_ (-0.4 g) and SO_2_ (-0.2 g). On the contrary, O_3_ was associated with a modest increase in birth weight (+0.9 g). Effects on births weight were observed also below WHO guideline values. When accounting for the prevailing exposure levels in Finland, CO was associated with the biggest reduction in birth weight. The effect of PM_2.5_ exposure on birthweight corresponds to a loss of 30 g at mean exposure. Assuming a causal relationship, about 700 cases of low birth weight could be attributable to PM_2.5_ in Finland during the study period.

**Conclusions:** No clear evidence on safe exposure level was found in this study. All pollutants were associated with reduced birthweight except ozone. Causality and confounding due to correlations warrant specific attention.

# Study population selection


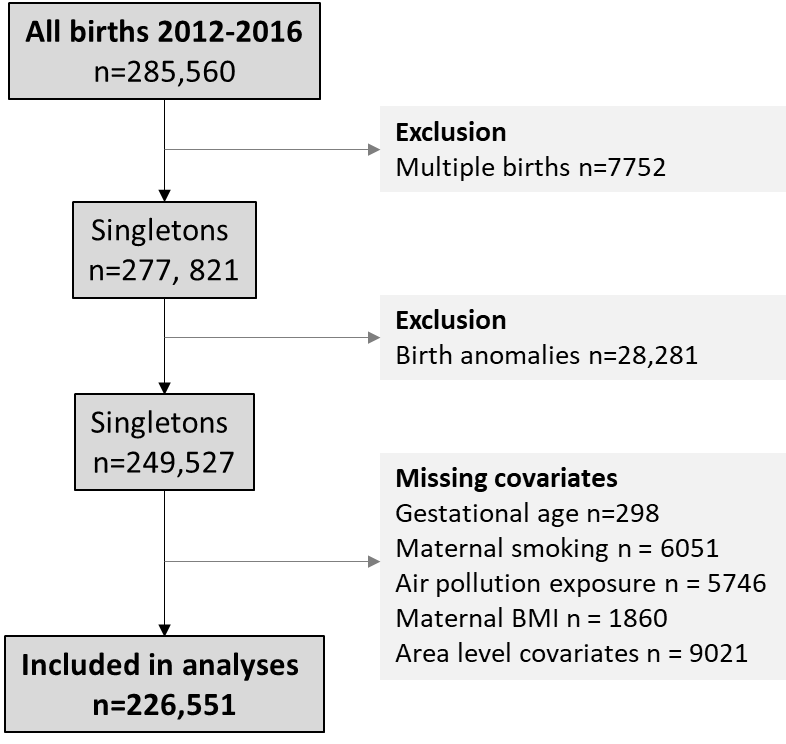


Figure S1. *Selection of the study population from all births in Finland (2012-2016) to singleton births without births anomalies and missing covariates.*

# Confounder selection

Table S1 summaries the selection of relevant confounder for the adjustment of the multivariate regression.

Table S1. *Univariate regressions between included confounders and birth weight (outcome) and air pollutants (exposures).*

| **Confounder** | **Birth weight** | | **PM_2.5_** | | **PM_10_** | | **PM_coarse_** | | **NO_2_** | | **NO_x_** | | **CO** | | **SO_2_** | | **O_3_** | |
| --- | --- | --- | --- | --- | --- | --- | --- | --- | --- | --- | --- | --- | --- | --- | --- | --- | --- | --- |
|  | **beta** | **p** | **beta** | **p** | **beta** | **p** | **beta** | **p** | **beta** | **p** | **beta** | **p** | **beta** | **p** | **beta** | **p** | **beta** | **p** |
| **Maternal age** | 2.80 | <0.01 | 0.02 | <0.01 | 0.05 | <0.01 | 0.03 | <0.01 | 0.22 | <0.01 | 0.45 | <0.01 | 0.28 | <0.01 | 0.33 | <0.01 | -0.1 | <0.01 |
| **Sex** (ref: boys) | -103.81 | <0.01 | -0.01 | 0.06 | -0.03 | 0.04 | -0.02 | <0.01 | -0.12 | 0.02 | -0.22 | 0.02 | -0.17 | 0.06 | -0.18 | 0.05 | 0.07 | 0.07 |
| **Season** (ref: spring) | | | | | | | | | | | | | | | | | | |
| Summer | 3.54 | 0.22 | 0.56 | <0.01 | 1.05 | <0.01 | 0.49 | <0.01 | 0.69 | <0.01 | 1.73 | <0.01 | 13.54 | <0.01 | 2.51 | <0.01 | -0.69 | <0.01 |
| Autumn | 5.95 | 0.04 | 0.61 | <0.01 | 1.09 | <0.01 | 0.49 | <0.01 | 1.07 | <0.01 | 2.26 | <0.01 | 17.02 | <0.01 | 3.22 | <0.01 | 3.21 | <0.01 |
| Winter | 10.27 | 0.00 | 0.27 | <0.01 | 0.42 | <0.01 | 0.15 | <0.01 | 0.8 | <0.01 | 1.52 | <0.01 | 8.26 | <0.01 | 1.55 | <0.01 | 3.69 | <0.01 |
| **Gestational age** | 23.87 | <0.01 | 0.00 | <0.01 | 0.01 | <0.01 | 0.006 | <0.01 | 0.04 | <0.01 | 0.08 | <0.01 | 0.007 | <0.01 | 0.05 | <0.01 | -0.02 | <0.01 |
| **SES** (ref: upper white collar) | | | | | | | | | | | | | | | | | | |
| Lower white collar | 3.08 | 0.41 | -0.40 | <0.01 | -1.03 | <0.01 | -0.64 | <0.01 | -4.52 | <0.01 | -8.56 | <0.01 | -6.79 | <0.01 | -7.35 | <0.01 | 2.69 | <0.01 |
| Blue collar | -14.19 | 0.00 | -0.45 | <0.01 | -1.19 | <0.01 | -0.75 | <0.01 | -5.16 | <0.01 | -9.73 | <0.01 | -8.37 | <0.01 | -8.37 | <0.01 | 3.27 | <0.01 |
| Other | -18.85 | 0.00 | -0.31 | <0.01 | -0.73 | <0.01 | -0.42 | <0.01 | -2.99 | <0.01 | -5.82 | <0.01 | -4.8 | <0.01 | -3.93 | <0.01 | 1.52 | <0.01 |
| Missing | -19.87 | 0.00 | -0.05 | <0.01 | 0.03 | 0.12 | 0.08 | <0.01 | 0.77 | <0.01 | 1.39 | <0.01 | 1.14 | <0.01 | -1.44 | <0.01 | -0.65 | <0.01 |
| **Nulliparous** (ref: multiparous) | -150.57 | <0.01 | 0.25 | <0.01 | 0.65 | <0.01 | 0.4 | <0.01 | 2.89 | <0.01 | 5.26 | <0.01 | 4.08 | <0.01 | 4.75 | <0.01 | -1.7 | <0.01 |
| **BMI** (ref: underweight) | | | | | | | | | | | | | | | | | | |
| normal weight | 169.69 | <0.01 | -0.08 | <0.01 | -0.21 | <0.01 | -0.13 | <0.01 | -0.75 | <0.01 | -1.29 | <0.01 | -1.2 | <0.01 | -1.01 | <0.01 | 0.58 | <0.01 |
| overweight | 250.71 | <0.01 | -0.24 | <0.01 | -0.65 | <0.01 | -0.41 | <0.01 | -2.71 | <0.01 | -4.98 | <0.01 | -4 | <0.01 | -4.37 | <0.01 | 1.85 | <0.01 |
| obese | 278.33 | <0.01 | -0.35 | <0.01 | -0.95 | <0.01 | -0.59 | <0.01 | -3.99 | <0.01 | -7.36 | <0.01 | -6.25 | <0.01 | -6.38 | <0.01 | 2.73 | <0.01 |
| **Maternal smoking** (ref: no smoking) | | | | | | | | | | | | | | | | | | |
| quit smoking during 1st trimester | -8.55 | 0.03 | -0.24 | <0.01 | -0.59 | <0.01 | -0.35 | <0.01 | -2.47 | <0.01 | -4.66 | <0.01 | -4.03 | <0.01 | -2.67 | <0.01 | 1.32 | <0.01 |
| Continued smoking after 1st trimester | -170.14 | <0.01 | -0.17 | <0.01 | -0.53 | <0.01 | -0.36 | <0.01 | -2.48 | <0.01 | -4.87 | <0.01 | -3.41 | <0.01 | -4.16 | <0.01 | 1.52 | <0.01 |
| **Area level** | | | | | | | | | | | | | | | | | | |
| Upper secondary education | 9.58 | 0.01 | 0.66 | <0.01 | 1.22 | <0.01 | 0.56 | <0.01 | 3.99 | <0.01 | 7.07 | <0.01 | 11.14 | <0.01 | 7.21 | <0.01 | -4.15 | <0.01 |
| Lowest income quartile | -111.44 | <0.01 | -4.39 | <0.01 | -9.38 | <0.01 | -4.99 | <0.01 | -29.43 | <0.01 | -54.46 | <0.01 | -61.44 | <0.01 | -23.9 | <0.01 | 9.74 | <0.01 |
| **Postal code area** (ref: region 0) | | | | | | | | | | | | | | | | | | |
| 1 | 17.12 | <0.01 | -1.51 | <0.01 | -4.26 | <0.01 | -2.75 | <0.01 | -17.13 | <0.01 | -33.57 | <0.01 | -26.56 | <0.01 | -21.97 | <0.01 | 11.19 | <0.01 |
| 2 | 22.41 | <0.01 | -1.13 | <0.01 | -2.99 | <0.01 | -1.86 | <0.01 | -16.16 | <0.01 | -32.01 | <0.01 | -31.14 | <0.01 | -15.2 | <0.01 | 13.44 | <0.01 |
| 3 | 22.40 | <0.01 | -1.85 | <0.01 | -4.61 | <0.01 | -2.76 | <0.01 | -18.24 | <0.01 | -35.4 | <0.01 | -28.35 | <0.01 | -28.08 | <0.01 | 10.92 | <0.01 |
| 4 | 9.71 | 0.02 | -1.77 | <0.01 | -4.36 | <0.01 | -2.59 | <0.01 | -17.88 | <0.01 | -34.67 | <0.01 | -29.05 | <0.01 | -22.74 | <0.01 | 9.12 | <0.01 |
| 5 | 17.96 | <0.01 | -1.88 | <0.01 | -4.92 | <0.01 | -3.04 | <0.01 | -18.99 | <0.01 | -35.25 | <0.01 | -30.32 | <0.01 | -27.94 | <0.01 | 8.54 | <0.01 |
| 6 | 62.35 | <0.01 | -2.64 | <0.01 | -5.52 | <0.01 | -2.88 | <0.01 | -22.15 | <0.01 | -40.01 | <0.01 | -42.26 | <0.01 | -28.22 | <0.01 | 13.85 | <0.01 |
| 7 | 6.52 | 0.22 | -2.90 | <0.01 | -6.28 | <0.01 | -3.38 | <0.01 | -20.59 | <0.01 | -37.77 | <0.01 | -37.62 | <0.01 | -16.09 | <0.01 | 6.81 | <0.01 |
| 8 | 6.15 | 0.19 | -2.98 | <0.01 | -6.42 | <0.01 | -3.45 | <0.01 | -22.86 | <0.01 | -41.27 | <0.01 | -40.53 | <0.01 | -25.73 | <0.01 | 8.08 | <0.01 |
| 9 | 7.79 | 0.03 | -2.61 | <0.01 | -4.59 | <0.01 | -1.97 | <0.01 | -18.19 | <0.01 | -33.09 | <0.01 | -32.14 | <0.01 | -13.04 | <0.01 | 5.47 | <0.01 |

# Air pollution exposure distribution

Table S2 shows distributional parameters of the residential outdoor air exposure levels during pregnancy. All means are slightly to clearly larger than medians, indicating slight to strong skewness, except for ozone, which has higher concentrations in rural areas and almost identical mean and median values.

Table S2*. Descriptives residential air pollution exposures (total pregnancy) in µg/m^3^*

|  | **Mean** | **Standard deviation (sd)** | **Minimum** | **25^th^ percentile** | **Median** | **75^th^ percentile** | **Maximum** |
| --- | --- | --- | --- | --- | --- | --- | --- |
| **Particles** | | | | | | | |
| **PM_2.5_** | 4.69 | 1.37 | 1.33 | 3.61 | 4.55 | 5.678 | 18.95 |
| **PM_10_** | 8.06 | 3.09 | 2.5 | 5.72 | 7.35 | 10 | 45.82 |
| **PM_coarse_** | 3.37 | 1.84 | 0.48 | 1.98 | 2.87 | 4.417 | 27.01 |
| **Gases** | | | | | | | |
| **NO_2_** | 13.99 | 11.95 | 0.35 | 5.11 | 9.78 | 18.98 | 72.18 |
| **NO_x_** | 21.28 | 22.93 | 0.38 | 5.75 | 12.02 | 27.29 | 159.15 |
| **CO** | 148 | 22.0 | 97.7 | 131 | 144 | 161 | 241 |
| **SO_2_** | 16.06 | 21.77 | 0.08 | 1.94 | 6.54 | 23.41 | 273.53 |
| **O_3_** | 52.33 | 8.46 | 20.19 | 47.5 | 53.96 | 58.43 | 75.49 |

# Categorical quartile analyses

We conducted a quartile comparison using the lowest quartile as reference (Table S3, Figure S2). For all pollutants a clear increase in reduction of birth weight was observed with increasing exposure quartile, except for ozone, for which an increase was observed. While the results were sensitive to the adjustment model, a clear exposure response remained in all models. However, in the higher adjustment models (models 3 and 4) the change in birth weight in the second quartile compared to the first quartile is not statistically significant for PM_2.5_. The association between SO_2_ and birth weight does not appear linear with non-linearity in the estimates for quartiles 2 and 3.


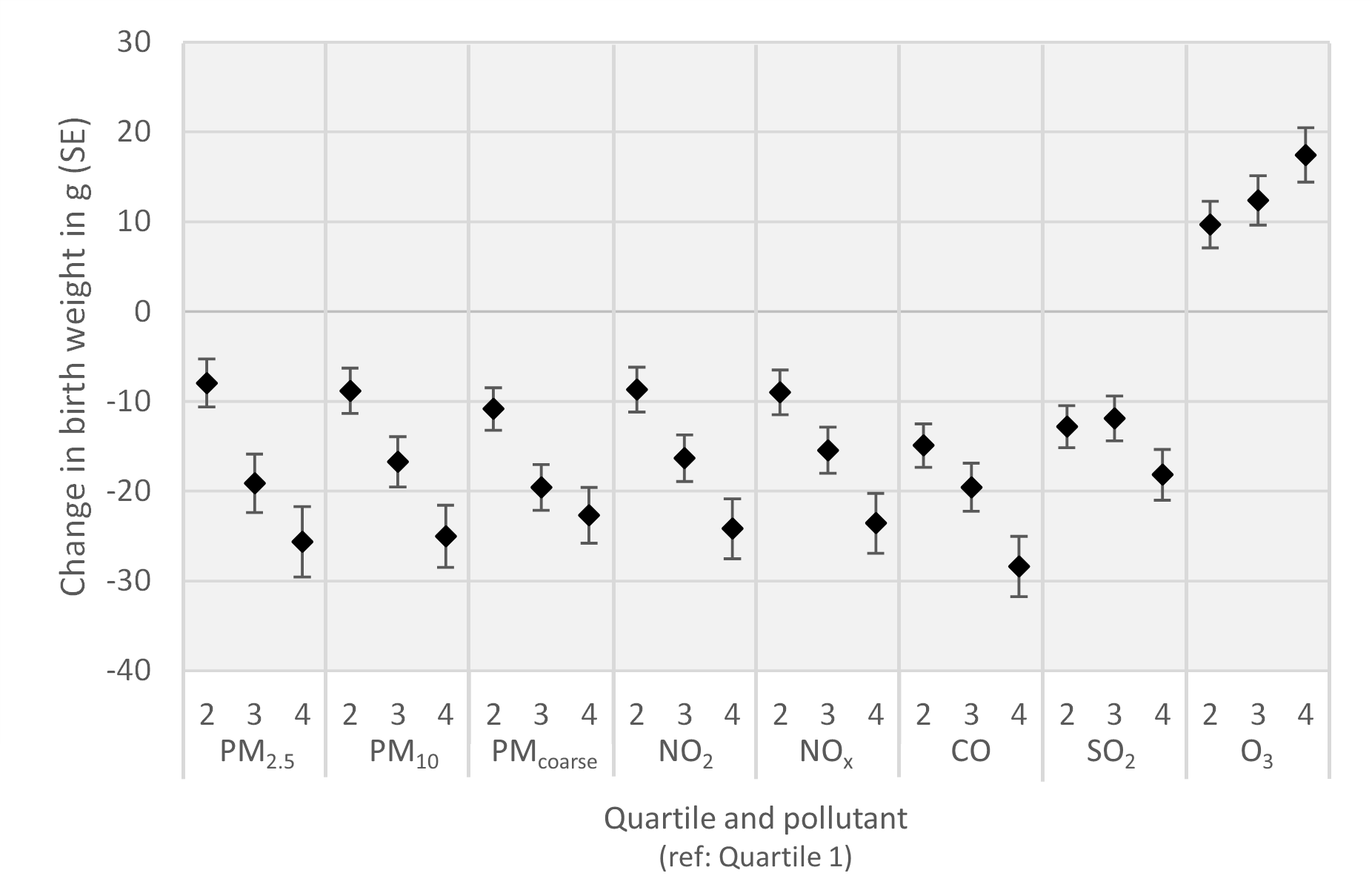


Figure S2. *Differences in birth weight between exposure quartiles comparison with lowest quartile as reference. For p values consult Table S3. Adjusted for gestational age (days), maternal age (years), sex, season, maternal socioeconomic status, parity, maternal pre-pregnancy BMI, maternal smoking, lowest Income quintile (postal code level), fraction of secondary education (postal code level), and region.*

Table S3*. Difference in birth weight between exposure quartiles (lowest quartile as reference). All differences are statistically significant at α = 0.005 unless marked otherwise.*

| **Pollutant** | **Quartile** | **crude** | | **Model 1^a^** | | **Model 2^b^** | | **Model 3^c^** | |
| --- | --- | --- | --- | --- | --- | --- | --- | --- | --- |
|  |  | **beta** | **p value** | **beta** | **p value** | **beta** | **p value** | **beta** | **p value** |
| **PM_2.5_**  (ref 1.33-3.61 µg/m^3^) | **2** (3.61-4.55 µg/m^3^) | -2.954 | 0.326* | -13.48 | <0.001 | 2.14 | 0.378* | -0.03 | 0.99* |
|  | **3** (4.55-5.68 µg/m^3^) | -14.653 | <0.001 | -37.33 | <0.001 | -11.84 | <0.001 | -15.46 | <0.001 |
|  | **4** (5.68-18.95 µg/m^3^) | -34.344 | <0.001 | -72.64 | <0.001 | -28.98 | <0.001 | -36.40 | <0.001 |
| **PM_10_**  (ref 2.5-5.72 µg/m^3^) | **2** (5.72-7.35 µg/m^3^) | -8.6 | 0.004 | -17.28 | <0.001 | -1.45 | 0.551* | -4.75 | 0.053* |
|  | **3** (7.35-10.00 µg/m^3^) | -23.57 | <0.001 | -39.87 | <0.001 | -10.93 | <0.001 | -14.75 | <0.001 |
|  | **4** (10.00-45.82 µg/m^3^) | -42.91 | <0.001 | -79.34 | <0.001 | -32.80 | <0.001 | -39.64 | <0.001 |
| **PM_coarse_**  (ref 0.48-1.98 µg/m^3^) | **2** (1.98-2.87 µg/m^3^) | -11.495 | <0.001 | -18.87 | <0.001 | -4.77 | 0.049* | -8.11 | 0.001 |
|  | **3** (2.87-4.42 µg/m^3^) | -31.44 | <0.001 | -42.22 | <0.001 | -12.60 | <0.001 | -15.67 | <0.001 |
|  | **4** (4.42-27.01 µg/m^3^) | -47.049 | <0.001 | -78.67 | <0.001 | -32.81 | <0.001 | -38.48 | <0.001 |
| **NO_2_**  (ref 0.25-5.11 µg/m^3^) | **2** (5.11-9.78 µg/m^3^) | -17.268 | <0.001 | -22.89 | <0.001 | -7.31 | 0.002 | -11.52 | <0.001 |
|  | **3** (9.78-18.98 µg/m^3^) | -36.832 | <0.001 | -48.19 | <0.001 | -17.27 | <0.001 | -20.67 | <0.001 |
|  | **4** (18.98-72.18 µg/m^3^) | -51.794 | <0.001 | -87.55 | <0.001 | -37.91 | <0.001 | -44.21 | <0.001 |
| **NO_x_**  (ref: 0.38-5.75 µg/m^3^) | **2** (5.75-12.02 µg/m^3^) | -16.146 | <0.001 | -22.84 | <0.001 | -7.38 | 0.002 | -11.79 | <0.001 |
|  | **3** (12.02-27.29 µg/m^3^) | -37.485 | <0.001 | -47.55 | <0.001 | -16.20 | <0.001 | -19.68 | <0.001 |
|  | **4** (27.29-159.15 µg/m^3^) | -49.929 | <0.001 | -86.62 | <0.001 | -37.73 | <0.001 | -44.49 | <0.001 |
| **CO**  (ref 97.66-131.00 µg/m^3^) | **2** (133.00-143.52 µg/m^3^) | -10.183 | <0.001 | -28.70 | <0.001 | -14.36 | <0.001 | -16.94 | <0.001 |
|  | **3** (143.52-161.30 µg/m^3^) | -27.614 | <0.001 | -53.14 | <0.001 | -23.37 | <0.001 | -26.76 | <0.001 |
|  | **4** (161.30-241.04 µg/m^3^) | -46.606 | <0.001 | -86.94 | <0.001 | -41.47 | <0.001 | -48.36 | <0.001 |
| **SO_2_**  (ref 0.08-1.94 µg/m^3^) | **2** (1.94-6.54 µg/m^3^) | -13.945 | <0.001 | -22.44 | <0.001 | -10.33 | <0.001 | -13.86 | <0.001 |
|  | **3** (6.54-23.41 µg/m^3^) | -24.031 | <0.001 | -37.53 | <0.001 | -15.22 | <0.001 | -19.51 | <0.001 |
|  | **4** (23.41-273.53 µg/m^3^) | -46.799 | <0.001 | -77.22 | <0.001 | -32.40 | <0.001 | -36.95 | <0.001 |
| **O_3_**  (ref 20.19-47.49 µg/m^3^) | **2** (47.49-53.96 µg/m^3^) | 22.439 | <0.001 | 46.94 | <0.001 | 21.46 | <0.001 | 22.58 | <0.001 |
|  | **3** (53.96-58.43 µg/m^3^) | 47.487 | <0.001 | 62.77 | <0.001 | 28.97 | <0.001 | 31.30 | <0.001 |
|  | **4** (58.43-75.49 µg/m^3^) | 53.07 | <0.001 | 81.45 | <0.001 | 40.29 | <0.001 | 43.23 | <0.001 |

*^a^ adjusted for gestational age (days), maternal age (years), sex, season*

*^b^ adjusted for Model 1 and maternal socioeconomic status, parity, maternal pre-pregnancy BMI, maternal smoking*

*^c^ adjusted for Model 2 and lowest Income quintile (postal code level), fraction of secondary education (postal code level)*

** Not statistically significant*

# Sensitivity analyses

As sensitivity analyses, we explored the potential effect of exclusion of preterm births, modification by an exposure threshold, and extension of the cohort to include birth between 1991 and 2011 (beyond the area level SES variable data availability). In the first sensitivity analysis we excluded all newborns born before 37 weeks of gestation assuming that the gestational age, especially in preterm birth, is the main determinant of (low) birth weight and thus potentially masking other, weaker determinants, such as air pollution exposure. Sensitivity to threshold was tested by excluding the lowest proportion of exposure (mean of 5^th^ percentile minus minimum) in the regression model, assuming that the exposure misclassification is highest in the tails.

Table S4. *Sensitivity analyses comparing different cohort definitions. Main: main analyses presented throughout the paper; term: only term birth included (>36 weeks gestation); above threshold: exclusion of the lowest exposure part (mean of minimum and 5^th^ percentile; inclusion of region indicator: in addition adjusted for national region Model 1: adjusted for gestational age (days), maternal age (years), sex, season; Model 2: adjusted for Model 1 and maternal socioeconomic status, parity, maternal pre-pregnancy BMI, maternal smoking; Model 3: adjusted for Model 2 and lowest Income quintile (postal code level), fraction of secondary education (postal code level).*

|  | **ORs per 1µg/m^3^** | | | | | | | | | | | | | | | |
| --- | --- | --- | --- | --- | --- | --- | --- | --- | --- | --- | --- | --- | --- | --- | --- | --- |
|  | **NO_x_** | | **NO_2_** | | **O_3_** | | **CO** | | **SO_2_** | | **PM_10_** | | **PM_2.5_** | | **PM_coarse_** | |
| Threshold cutoff (µg/m^3^) | 1.34 | | 1.24 | | 28.43 | | 107.98 | | 0.31 | | 3.32 | | 2.04 | | 0.83 | |
|  | **Beta** | **±SE** | **Beta** | **±SE** | **Beta** | **±SE** | **Beta** | **±SE** | **Beta** | **±SE** | **Beta** | **±SE** | **Beta** | **±SE** | **Beta** | **±SE** |
| **Crude model** | | | | | | | | | | | | | | | | |
| Main | -0.80 | 0.05 | -1.61 | 0.09 | 2.44 | 0.13 | -0.76 | 0.05 | -0.76 | 0.05 | -5.21 | 0.34 | -9.12 | 0.78 | -9.66 | 0.58 |
| Term BW | -0.82 | 0.04 | -1.64 | 0.08 | 2.55 | 0.12 | -0.78 | 0.04 | -0.78 | 0.04 | -5.22 | 0.32 | -8.84 | 0.72 | -9.86 | 0.53 |
| Above threshold | -0.69 | 0.04 | -1.65 | 0.09 | 2.48 | 0.13 | -0.81 | 0.05 | -0.77 | 0.05 | -5.44 | 0.35 | -9.55 | 0.79 | -9.74 | 0.58 |
| **Model 1** | | | | | | | | | | | | | | | | |
| Main | -1.43 | 0.04 | -2.84 | 0.07 | 3.78 | 0.11 | -1.46 | 0.04 | -1.23 | 0.04 | -9.69 | 0.29 | -20.05 | 0.66 | -16.25 | 0.49 |
| Term BW | -1.38 | 0.04 | -2.72 | 0.08 | 3.72 | 0.11 | -1.41 | 0.04 | -1.19 | 0.04 | -9.29 | 0.29 | -18.91 | 0.67 | -15.76 | 0.49 |
| Above threshold | -1.29 | 0.04 | -2.86 | 0.07 | 3.82 | 0.11 | -1.47 | 0.04 | -1.22 | 0.04 | -9.72 | 0.29 | -20.30 | 0.67 | -16.24 | 0.49 |
| **Model 2** | | | | | | | | | | | | | | | | |
| Main | -0.61 | 0.04 | -1.22 | 0.07 | 1.78 | 0.11 | -0.70 | 0.04 | -0.44 | 0.04 | -4.00 | 0.29 | -8.16 | 0.65 | -6.75 | 0.48 |
| Term BW | -0.58 | 0.04 | -1.15 | 0.08 | 1.76 | 0.11 | -0.66 | 0.04 | -0.42 | 0.04 | -3.73 | 0.29 | -7.32 | 0.66 | -6.47 | 0.49 |
| Above threshold | -0.62 | 0.04 | -1.25 | 0.08 | 1.87 | 0.11 | -0.70 | 0.04 | -0.44 | 0.04 | -4.06 | 0.29 | -8.38 | 0.66 | -6.75 | 0.49 |
| **Model 3** | | | | | | | | | | | | | | | | |
| Main | -0.69 | 0.04 | -1.37 | 0.08 | 1.92 | 0.11 | -0.82 | 0.04 | -0.47 | 0.04 | -4.79 | 0.30 | -10.18 | 0.68 | -7.80 | 0.50 |
| Term BW | -0.62 | 0.04 | -1.25 | 0.07 | 1.77 | 0.10 | -0.77 | 0.04 | -0.44 | 0.04 | -4.45 | 0.29 | -9.51 | 0.65 | -7.21 | 0.47 |
| Above threshold | -0.68 | 0.04 | -1.40 | 0.08 | 2.02 | 0.11 | -0.83 | 0.04 | -0.47 | 0.04 | -4.84 | 0.30 | -10.41 | 0.69 | -7.77 | 0.50 |
| Inclusion of region indicator | -0.33 | 0.05 | -0.75 | 0.10 | 0.80 | 0.13 | -0.49 | 0.06 | -0.16 | 0.05 | -2.84 | 0.41 | -6.35 | 1.0 | -4.4 | 0.62 |

As part of the sensitivity analyses, we also tested for sensitivity of the observed risk estimate to changed in the covariate definition and alternative adjustment models. Assuming Model 3 in the manuscript as the main model, we replaced continuous maternal age with a categorised variable and gestational age as weeks.

Table S5. *Categorical variable for maternal age.*

| **Age (years)** | **<20** | **21-24** | **25-29** | **30-34** | **35-39** | **40-44** | **>44** |
| --- | --- | --- | --- | --- | --- | --- | --- |
| **Count** | 4135 | 32878 | 68924 | 75108 | 37263 | 7767 | 476 |

Table S6. *Main analyses (adjustment model 3) and modified adjustment for categorical maternal age (instead of continuous) and gestational week (instead of gestational age in days). Changes in birth weight (in g) per IQR increment in exposure.*

|  | **IQR** | **Model 3^a^** | | **Modified Model 3^b^** | |
| --- | --- | --- | --- | --- | --- |
|  | **µg/m3** | **beta** | **se** | **beta** | **se** |
| **PM_2.5_** | 2.07 | -21.1 | 1.41 | -21.5 | 1.34 |
| **PM_10_** | 4.28 | -21.0 | 1.28 | -20.0 | 1.24 |
| **PM_crs_** | 2.44 | -19.0 | 1.22 | -17.9 | 1.17 |
| **NO_2_** | 13.87 | -19.0 | 1.11 | -18.5 | 0.97 |
| **NO_x_** | 21.54 | -14.9 | 0.86 | -14.2 | 0.86 |
| **CO** | 30.00 | -24.6 | 1.20 | -24.0 | 1.20 |
| **SO_2_** | 21.47 | -10.1 | 0.86 | -9.7 | 0.86 |
| **O_3_** | 10.93 | 21.0 | 1.2 | 19.7 | 1.09 |

*^a^ adjusted for gestational age (days), maternal age (years), sex, season, maternal socioeconomic status, parity, maternal pre-pregnancy BMI, maternal smoking, lowest Income quintile (postal code level), fraction of secondary education (postal code level)*

*^b^ adjusted for gestational age (weeks), maternal age (categorical), sex, season, maternal socioeconomic status, parity, maternal pre-pregnancy BMI, maternal smoking, lowest Income quintile (postal code level), fraction of secondary education (postal code level)*

# Threshold analyses

We tested for a potential threshold by restricting the study population to those pregnancies, which were exposed to lower levels than those recommended in the WHO guidelines (WHO, 2021). For those pollutants, where no annual guideline values were available, we derived values based on expert judgement: for scaling from short-term to long-term exposure (CO, SO2), we divided the short-term value by 2.5. For NO_x_ we assumed double value as for NO_2_. For PM_crs_ we applied the same value as for PM_2.5_ due to comparable exposure levels. Lastly, we applied the peak-season value for O_3_ as cut-off value.

**Table S7.** *Main analyses (adjustment model 3) testing for threshold.*

|  | | **Change in birth weight per 1 µg/m^3^ increment in exposure ^a^** | | | | | | |  |
| --- | --- | --- | --- | --- | --- | --- | --- | --- | --- |
|  |  | **Whole population** | | **WHO guideline subpopulation** | | | | | **Beta ratio^c^** |
| **Pollutant** | **Cut-off definition** | **beta** | **se** | **Cut-off [µg/m^3^]** | **Median [µg/m^3^]** | **n** | **beta** | **se** |  |
| **PM_2.5_** | Guideline | -10.18 | 0.68 | 5 | 3.8 | 139,052 | -3.28 | 1.48 | 0.32 |
| **PM_10_** | Guideline | -4.79 | 0.3 | 15 | 7.3 | 221,447 | -5.41 | 0.28 | 1.13 |
| **PM_crs_** | Expert judgement | -7.80 | 0.5 | 5 | 2.5 | 181,345 | -7.29 | 0.91 | 0.93 |
| **NO_2_** | Guideline | -1.37 | 0.08 | 10 | 5.2 | 115,200 | -2.82 | 0.55 | 2.06 |
| **NO_x_** | Expert judgement | -0.69 | 0.04 | 20 | 7.8 | 152,643 | -1.52 | 0.48 | 2.20 |
| **CO** | Estimated from daily^b^ | -0.82 | 0.04 | 1600 | 143.7 | 226,551 | -0.82 | 0.04 | 1.00 |
| **SO_2_** | Estimated from daily^b^ | -0.47 | 0.04 | 16 | 2.8 | 147,507 | -1.21 | 0.26 | 2.57 |
| **O_3_** | Peak season value | 1.92 | 0.11 | 60 | 52.3 | 188,029 | 1.82 | 0.12 | 0.95 |

*^a^ adjusted for gestational age (days), maternal age (years), sex, season, maternal socioeconomic status, parity, maternal pre-pregnancy BMI, maternal smoking, lowest Income quintile (postal code level), fraction of secondary education (postal code level)*

*^b^ daily limit value divided by 2.5*

*^c^ ratio of beta WHO guideline subpopulation divided by beta of whole population*

WHO, 2021. <https://www.who.int/news-room/feature-stories/detail/what-are-the-who-air-quality-guidelines> (accessed 29 November 2024)

# Impact assessment

We estimated the impact of air pollution exposure on birth weight by scaling the observed change in birth weight to the mean exposure in the study population.

Table S8. *Impact of mean air pollution exposure during pregnancy on birth weight.*

|  | **Mean exposure (µg/m^3^)** | **Impact** | | | | | | | |
| --- | --- | --- | --- | --- | --- | --- | --- | --- | --- |
|  |  | **crude** | | **Model 1^a^** | | **Model 2^b^** | | **Model 3^c^** | |
|  |  | **Change (g)** | **Ratio*** | **Change (g)** | **Ratio*** | **Change (g)** | **Ratio*** | **Change (g)** | **Ratio*** |
| **PM_2.5_** | 4.7 | -42.8 | 0.9 | -94.0 | 2.0 | -38.3 | 0.8 | -47.7 | 1.0 |
| **PM_10_** | 8.1 | -42.0 | 1.1 | -78.1 | 2.0 | -32.2 | 0.8 | -38.6 | 1.0 |
| **PM_crs_** | 3.4 | -32.6 | 1.2 | -54.8 | 2.1 | -22.7 | 0.9 | -26.3 | 1.0 |
| **NO_2_** | 14.0 | -22.6 | 1.2 | -39.7 | 2.1 | -17.1 | 0.9 | -19.2 | 1.0 |
| **NO_x_** | 21.3 | -17.1 | 1.2 | -30.5 | 2.1 | -13.1 | 0.9 | -14.6 | 1.0 |
| **CO** | 147.6 | -111.6 | 0.9 | -215.4 | 1.8 | -102.6 | 0.9 | -121.7 | 1.0 |
| **SO_2_** | 16.1 | -12.2 | 1.6 | -19.8 | 2.6 | -7.1 | 0.9 | -7.6 | 1.0 |
| **O_3_** | 52.3 | 127.4 | 1.3 | 197.8 | 2.0 | 93.1 | 0.9 | 100.6 | 1.0 |

** Ratio in reference to Model 3*

*^a^ adjusted for gestational age (days), maternal age (years), sex, season*

*^b^ adjusted for Model 1 and maternal socioeconomic status, parity, maternal pre-pregnancy BMI, maternal smoking*

*^c^ adjusted for Model 2 and lowest Income quintile (postal code level), fraction of secondary education (postal code level)*

*^d^ adjusted for Model 3 and national region indicator*

The gestational age, strongest known determinant of the birth weight, was adjusted for in all models 1-3. In generalised linear models the linear adjustment will omit any non-linearity of the gestational age-birth weight association (Figure S3). During gestational days from 200 to 240 the birth weight develops somewhat slower than during gestational days from 240 to 290. To avoid the residual effect due to this nonlinearity we considered transforming the independent variable from birth weight to deviance from the expected birth weight for each duration of gestation. The results were similar (data not shown) and to maintain consistency with the standard epidemiological approaches we chose to report birth weight models adjusted for gestational age.

Figure S3. *Observed birth weights by gestational age, linear fit showing the adjustment, 2SD whiskers, bottom of which shows the suggestive small for gestational age limit, and number of births by gestational age, which is a strong predictor of birth weight, but the association is slightly non-linear.*
